# Supplementation with the Symbiotic Formulation Prodefen^®^ Increases Neuronal Nitric Oxide Synthase and Decreases Oxidative Stress in Superior Mesenteric Artery from Spontaneously Hypertensive Rats

**DOI:** 10.3390/antiox11040680

**Published:** 2022-03-30

**Authors:** Pablo Méndez-Albiñana, Ángel Martínez-González, Laura Camacho-Rodríguez, Álvaro Ferreira-Lazarte, Mar Villamiel, Raquel Rodrigues-Díez, Gloria Balfagón, Ana B. García-Redondo, Mª Isabel Prieto-Nieto, Javier Blanco-Rivero

**Affiliations:** 1 Department of Physiology, School of Medicine, Universidad Autónoma de Madrid, 28029 Madrid, Spain; pablomendezalbi@gmail.com (P.M.-A.); mgangel9797@gmail.com (Á.M.-G.); lauri166@gmail.com (L.C.-R.); gloria.balfagon@uam.es (G.B.); ana.garcia@uam.es (A.B.G.-R.); 2 Group of Chemistry and Functionality of Carbohydrates and Derivatives, Food Science Research Institute (CIAL) (CSIC-UAM), 28049 Madrid, Spain; alvaro.ferreira@csic.es (Á.F.-L.); m.villamiel@csic.es (M.V.); 3 Department of Pharmacology and Therapeutics, School of Medicine, Universidad Autónoma de Madrid, 28029 Madrid, Spain; raquel.rodrigues@uam.es; 4 Research Institute University Hospital la Paz (IdIPaz), 28029 Madrid, Spain; 5 Center for Biomedical Research Network (CIBER) in Cardiovascular Diseases, 28029 Madrid, Spain; 6 Department of General and Digestive Surgery, Hospital Universitario la Paz, 28046 Madrid, Spain

**Keywords:** hypertension, synbiotic, perivascular mesenteric innervation, neuronal nitric oxide, protein kinase A, protein kinase C, PI3K-AKT, oxidative stress, Nrf2

## Abstract

In recent years, gut dysbiosis has been related to some peripheral vascular alterations linked to hypertension. In this work, we explore whether gut dysbiosis is related to vascular innervation dysfunction and altered nitric oxide (NO) production in the superior mesenteric artery, one of the main vascular beds involved in peripheral vascular resistance. For this purpose, we used spontaneously hypertensive rats, either treated or not with the commercial synbiotic formulation Prodefen^®^ (10^8^ colony forming units/day, 4 weeks). Prodefen^®^ diminished systolic blood pressure and serum endotoxin, as well as the vasoconstriction elicited by electrical field stimulation (EFS), and enhanced acetic and butyric acid in fecal samples, and the vasodilation induced by the exogenous NO donor DEA-NO. Unspecific nitric oxide synthase (NOS) inhibitor L-NAME increased EFS-induced vasoconstriction more markedly in rats supplemented with Prodefen^®^. Both neuronal NO release and neuronal NOS activity were enhanced by Prodefen^®^, through a hyperactivation of protein kinase (PK)A, PKC and phosphatidylinositol 3 kinase-AKT signaling pathways. The superoxide anion scavenger tempol increased both NO release and DEA-NO vasodilation only in control animals. Prodefen^®^ caused an increase in both nuclear erythroid related factor 2 and superoxide dismutase activities, consequently reducing both superoxide anion and peroxynitrite releases. In summary, Prodefen^®^ could be an interesting non-pharmacological approach to ameliorate hypertension.

## 1. Introduction

Hypertension is a complex disorder, characterized by maintained enhancement in both systolic and diastolic pressures (>130 mm Hg and >90 mm Hg, respectively) [1], and has a high prevalence, being estimated to cause 9.4 million deaths globally every year [2]. This pathology, influenced by both genetic and environmental factors, can be triggered by different pathologies, such as obesity, insulin resistance or hyperthyroidism, involving multiple organs and systems, and is the primary modifiable risk factor for heart disease [3,4,5].

The mesenteric vascular bed, located in the peritoneal cavity, belongs to the splanchnic vasculature, and plays a major role in generating and maintaining systemic vascular resistance. Mesenteric blood flow can constitute up to 20 to 30% of the total cardiac output and contributes to the redistribution of blood to the systemic circulation, consequently maintaining blood perfusion to different vital organs [6]. Arterial tone is regulated by myogenic mechanisms, endothelial and humoral factors, and perivascular innervation. Focusing on the latter, mesenteric vasculature has, amongst other factors, rich and functional nitrergic innervation, characterized by the release of NO, a vasodilator neurotransmitter that can reduce the maximum tone generated by noradrenaline by up to 50% [7,8]. Neuronal NO synthase (nNOS), a constitutive and Ca^2+^-dependent enzyme located in the nitrergic nerve endings, is sensitive to phosphorylation in the serine 1417 site, which determines activation of this enzyme and consequent synthesis of NO [9]. Previous reports from our group have shown enhanced neuronal NO release in hypertensive rats, due to protein kinase (PK)C activity, as an attempt to counteract increased vascular tone in this pathology [10,11]. In addition, other mechanisms, such as the phosphatidylinositol 3 kinase (PI3K)/AKT and the PKA signaling pathways, are also implicated in nNOS activation in the superior mesenteric artery of rats [12,13].

A pro-oxidative microenvironment appears in hypertension, leading to enhancement in vascular superoxide anion formation, which reduces neuronal NO bioavailability and thereby alters nitrergic function [14,15]. The organism contains multiple antioxidant defense mechanisms, which keep oxidative stress to appropriate levels. Transcription factor Nrf2 (nuclear factor erythroid 2-related factor 2) regulates adaptive response to oxidative stress. In situations in which reactive oxidative species levels are increased, Nrf2 translocates to the nucleus, promoting the transcription of different genes with an antioxidant role like superoxide dismutase (SOD), among others [16,17,18]. Diminished levels of Nrf2 and the antioxidant enzymes regulated by this transcription factor have been observed in hypertension [18].

Alterations in eating habits and lifestyle are undoubtedly the most important non-pharmacological interventions for the prevention and treatment of hypertension [5]. In this sense, our group has reported that a moderate aerobic exercise pattern restored the altered neuronal function observed in different hypertensive models [15,19]. However, this effect is limited and the design of more attractive approaches for patients is required. Gut microbiota (GM) are the community of living microorganisms that colonize the gastrointestinal tract, either permanently or temporarily [20]. Increasing evidence links the development of hypertension to dysbiosis, an imbalance in GM [21]. A supplementation with probiotics, live bacterial strains, has been shown to modulate the alteration of GM, as well as increase activity of various antioxidant enzymes [22,23,24], restoring NO to physiological levels. Multi-strain and/or multi-species probiotics have been shown, in animal models, to be more effective than a supplementation with mono-strain probiotics [25]. Additionally, the combination of probiotics with prebiotics, non-digestible oligosaccharides recently defined as “a substrate that is selectively utilized by host microorganisms conferring a health benefit” [26], is more effective than probiotics alone in improving survival and implantation of live microbes in the gastrointestinal tract [27]. The commercial synbiotic formulation Prodefen^®^ combines various probiotic strains (*Lactobacillus rhamnosus*, *Lactobacillus casei*, *Lactobacillus acidophilus*, *Lactobacillus bulgaricus*, *Streptococcus thermophilus*, *Bifidobacterium breve* and *Bifidobacterium infantis*) together with a prebiotic, fructooligosaccharides. Among the multiple beneficial effects produced by synbiotic agents, we have previously determined an antihypertensive role for the analogous commercial formulation Prodefen^®^ Plus, together with improvement of different metabolic syndrome symptoms, such as insulin resistance, hyperlipidemia, and liver steatosis, there is an improvement in perivascular nitrergic function [12].

Given the above, we hypothesize that the modulation of gut microbioma with the commercial synbiotic formula Prodefen^®^ might be an interesting non-pharmacological approach to ameliorate vascular disorders in hypertension. Our objective is to determine whether supplementation with the commercial synbiotic formulation Prodefen^®^ could improve neuronal NO release and vascular oxidative stress in mesenteric arteries from spontaneously hypertensive rats (SHR), and the possible mechanisms involved.

## 2. Materials and Methods

### 2.1. Animals

Male 4-month-old spontaneously hypertensive rats (SHR, *n* = 26) were raised and housed in the Animal Facility of the Universidad Autónoma de Madrid (Registration number EX-021U), held in pairs of 2 in appropriate cages, in controlled environmental conditions (20–24 °C, 55% relative humidity, 12-h light-dark cycle). The animals had access to fresh water and specific rat chow ad libitum.

Animals were randomly divided into two groups: (1) Control rats (SHR-Ctrl; *n* = 12); II) Rats supplemented with the synbiotic commercial formula Prodefen^®^ (10^8^ colony forming units (c.f.u.)/day, SHR-SYNB; *n* = 14) for 4 weeks. Some experiments were also performed in normotensive Wistar Kyoto (WKY) rats (*n* = 12, see Supplementary Materials).

The multi-strain synbiotic Prodefen^®^ (990 mg of fructooligosaccharides, 10^9^ c.f.u. of a mixture of: *Lactobacillus casei PXN 37*, *Lactobacillus rhamnosus PXN 54*, *Streptococcus thermophilus PXN 66*, *Bifidobacterium breve PXN 25*, *Lactobacillus acidophilus PXN 35*, *Bifidobacterium infantis PXN 27*, *Lactobacillus bulgaricus PXN 39*) was generously provided by Italfarmaco, S.A. This synbiotic formula was administered to the rats dissolved in drinking water. Regular water checking was performed to ensure the animals received the appropriate dose. The dose (10^8^ c.f.u./day) and administration time (4 weeks) of Prodefen^®^ was chosen based on previous pilot studies, choosing the lowest dose/time in which we found a systemic effect.

### 2.2. Blood Pressure Measurements

Systolic blood pressure (SBP) was measured in awake rats by a tail-cuff method (Letica, Digital Pressure Meter, LE5000, Barcelona, Spain) [15]. The measurements were performed before and after the supplementation was provided. All the animals were hypertensive at the beginning of the experimental protocol (SBP: 204.9 ± 3.36 mm Hg, *n* = 26).

### 2.3. Animal Euthanasia and Sample Collection

After overnight fasting, rats were euthanized by exsanguination by puncture of the infra-hepatic inferior cava vein, after anaesthesia (100 mg/kg ketamine hydrochloride, 12 mg/kg xylazine; i.m.). Blood samples were kept at room temperature for 2 h, and afterwards centrifuged (2000× *g*, 10 min, 4 °C). The supernatant (serum) was collected and kept at −70 °C until use. In addition, faecal samples were extracted from the caecum and were quickly frozen in liquid nitrogen and maintained at −70 °C until use.

The superior mesenteric artery was carefully dissected, cleaned of connective tissue, and maintained in cold (4 °C) Krebs–Henseleit solution (KHS) (in mmol/L: 115 NaCl, 25 NaHCO_3_, 4.7 KCl, 1.2 MgSO_4_·7H_2_O, 2.5 CaCl_2_, 1.2 KH_2_PO_4_,11.1 glucose, and 0.01 Na_2_EDTA) bubbled with a 95% O_2_–5% CO_2_ mixture. The endothelium was mechanically removed by gently rubbing the luminal surface of the segments with a thin wooden stick. This avoided possible actions by different drugs on endothelial cells that could lead to misinterpretation of the results. Some segments were quickly frozen in liquid nitrogen and maintained at −70 °C.

### 2.4. Circulating Endotoxin Levels

Circulating endotoxin levels were measured in serum samples from both experimental groups. For this purpose, the Pierce™ Chromogenic Endotoxin Quan Kit was used, following the manufacturer’s protocol (Thermo Fisher Scientific, Waltham, MA, USA). The results were expressed as Endotoxin Units (E.U.)/mL.

### 2.5. Short-Chain Fatty Acids (SCFA) Analysis

SCFA analysis was performed by liquid chromatography using an UV-975 detector, following the method described by Sanz et al. [28]. Briefly, faecal samples were filtered and injected on an HPLC system (Agilent Technologies, Frankfurt, Germany) equipped with an UV-975 detector and automatic injector. SCFA were separated using a Rezex ROA Organic Acids column (300 × 7.8 mm) (Phenomenex, Macclesfield, UK) thermostated at 50 °C. The mobile phase was sulfuric acid 0.005 mmol/L in HPLC grade water at a flow rate of 0.5 mL/min under isocratic elution. The elution profile was monitored at 210 nm and peaks were compared to standards to be identified. Data acquisition and integration were done using Agilent ChemStation software (Wilmington, DE, USA). Calibration curves of all SCFA were obtained from the analysis of standard solutions of lactic, formic, acetic, propionic and butyric acid. Results were expressed in mmol SCFA/L.

### 2.6. Vascular Reactivity

Isometric tension recording was measured in endothelium-denuded mesenteric segments from both SHR-Ctrl and SHR-SYNB, following the method described by Nielsen and Owman [29]. Functional integrity of the vessels was checked by exposing the segments to 75 mmol/L KCl (SHR-Ctrl: 9.25 + 0.7 mN; SHR-SYNB: 10.19 + 0.7 mN; *p* > 0.05). After a washout period, the absence of vascular endothelium was tested by the inability of 10 µmol/L acetylcholine (ACh) to relax segments precontracted with noradrenaline (NA).

Frequency-response curves to electrical field stimulation (EFS) were performed. The parameters used for EFS were 200 mA, 0.3 ms, 1–16 Hz, for 30 s with an interval of 1 min between each stimulus, the time required to recover basal tone. To analyze the participation of NO in the EFS-induced response in our experimental procedure, 0.1 mmol/L Nω-nitro-l-arginine methyl ester (L-NAME), a non-specific inhibitor of nitric oxide synthase (NOS), was added to the bath 30 min before performing the second frequency–response curve. A washout period of at least 1 h was necessary to avoid desensitization between consecutive curves.

Vasodilator response to the NO donor, diethylamine NONOate, (DEA-NO, 0.1 nmol/L–0.1 mmol/L) was determined in NA-precontracted segments from the two groups. Some segments were preincubated with tempol, to determine the potential role of oxidative stress in this response.

### 2.7. Nitric Oxide Release

NO release was measured in endothelium-denuded mesenteric segments from both experimental groups, using the fluorescent probe 4,5-diaminofluorescein (DAF-2) [15]. Some segments were incubated with 1 µmol/L H89 (a PKA inhibitor), 0.1 µmol/L calphostin C (a PKC inhibitor), 10 µmol/L LY 294002 (a PI3K inhibitor), or 0.1 mmol/L tempol (a superoxide anion scavenger) to determine the modulatory effect of these drugs on NO release. The modulatory effect of these drugs was calculated either by subtracting NO release after preincubation with the different inhibitors from that evoked in conditions without inhibitors; or by calculating the percentage of inhibition produced by each drug. The amount of NO released was expressed as arbitrary fluorescence units/mg tissue.

### 2.8. Detection of Superoxide Anions

Superoxide anion levels were measured in mesenteric rings without endothelium from both SHR-Ctrl and SHR-SYNB animals, by using the chemiluminiscent probe lucigenin, as previously described [30]. Blank samples (HEPES + lucigenin without arterial segment) were collected in the same way from the culture medium to subtract background emission.

### 2.9. Peroxynitrite Detection

The fluorescent probe dihydrorhodamine 123 (DHR) was used to determine peroxynitrite levels in de-endothelized arteries from both experimental groups, as previously described [30]. The amount of peroxynitrite released was expressed as arbitrary fluorescence units/mg tissue.

### 2.10. Superoxide Dismutase Activity

Frozen mesenteric segments without endothelium were homogenized in ice cold 0.1 mmol/L Tris-HCl, pH 7.4, solution, containing 0.5% Triton X-100, 5 mmol/L β-mercaptoethanol and 0.1 mg/mL PMSF. After centrifugation at 14,000× *g* (5 min, 4 °C), 20 μL of supernatants were used in the assay. Enzyme activity was measured using a Superoxide Dismutase Activity Assay Kit (Colorimetric) (Abcam, Cambridge, UK). Following the manufacturer’s instructions, the superoxide dismutase activity was expressed as a percentage of inhibition [31].

### 2.11. PKA and PKC Activity Assays

PKA and PKC activities were respectively determined using a PKA kinase activity assay kit or a PKC kinase activity assay kit (Abcam, Cambridge, UK), following the manufacturers’ protocols [12].

### 2.12. Western Blot Analysis

Western blot analysis was performed as previously described [15]. Frozen segments without endothelium were homogenized, and 30 µg protein were loaded in each lane. Mouse monoclonal antibody against nNOS (1:2000), rabbit polyclonal anti-nNOS (neuronal) (phospho S1417) antibody (1:2000), rabbit polyclonal anti-PI 3 Kinase p85 beta antibody (1:500), rabbit polyclonal anti-pan-AKT antibody (1:1000), rabbit polyclonal anti-pan-AKT (phospho T308) antibody (1:500), rabbit polyclonal anti-superoxide dismutase 1 antibody (1:500), mouse monoclonal superoxide dismutase 2 antibody (1:1000), rabbit polyclonal anti-Nrf2 antibody (1:1000) and rabbit polyclonal Phospho-Nrf2 (Ser 40) antibody (1:1000) were used. Appropriate secondary antibodies were used (1:2000). The development and quantification of the images were performed using Quantity One software (v. 4.6.6, Biorad, Madrid, Spain). The same membrane was used to correct protein expression in each sample, by means of a monoclonal anti-β-actin−peroxidase antibody (1:50,000).

### 2.13. Drugs and Antibodies Used

The different drugs were purchased from Sigma-Aldrich (Madrid, Spain), except for LY294002 and H89, obtained from Tocris (Bristol, UK). Distilled water, dimethyl sulfoxide, or a NaCl (0.9%)-ascorbic acid (0.01% *w*/*v*) solution were used to make stock solutions (10 mmol/L), which were kept at −20 °C. Appropriate dilutions were made in KHS on the day of the experiment. The different vehicles did not affect basal tone.

Mouse monoclonal antibody against nNOS was purchased from BD Biosciences (Spain), rabbit polyclonal anti-nNOS (neuronal) (phospho S1417) antibody, rabbit polyclonal anti-PI 3 Kinase p85 beta antibody, rabbit polyclonal anti-pan-AKT antibody, rabbit polyclonal anti-pan-AKT (phospho T308) antibody, rabbit polyclonal anti-superoxide dismutase 1 antibody and rabbit polyclonal anti-Nrf2 antibody were purchased from Abcam (Cambridge, UK), mouse monoclonal superoxide dismutase 2 antibody was purchased from Santa Cruz Biotechnology (Santa Cruz, CA, USA), rabbit polyclonal Phospho-Nrf2 (Ser 40) antibody was purchased from Thermo Fisher Scientific (Waltham, MA, USA); and monoclonal anti-β-actin−peroxidase antibody was purchased from Sigma-Aldrich (Spain). Anti-mouse and anti-rabbit secondary antibodies were purchased from GE Healthcare Systems (Chicago, IL, USA).

### 2.14. Data Analysis

Graph representation and statistical analysis were performed using GraphPad Prism 8.0 software (San Diego, CA, USA). The responses induced by EFS were expressed as a percentage of the initial contraction elicited by 75 mmol/L KCl for comparison between experimental groups. To determine differences in the effect of preincubation with the different drugs in EFS-induced contraction experiments, we analysed the differences between areas under the curve (dAUC). The relaxation induced by DEA-NO was expressed as a percentage of the initial contraction elicited by NA. Results were expressed as mean ± S.E.M. The EFS or DEA-NO vasomotor responses were compared by means of an unpaired two-way analysis of variance (ANOVA). When comparing the effect of L-NAME on EFS-induced contraction, we used a paired two-way ANOVA. For systolic blood pressure, endotoxin levels, SCFA levels, KCl, dAUC, NO release, superoxide anion and peroxynitrite formation, SOD activity, PKA activity, PKC activity and Western Blot analyses, the ROUT method was used to identify and remove outliers. Moreover, we applied a Saphiro-Wilk test to check the normality of the population data and, afterwards, we used a Student *t*-test. *p* < 0.05 was considered significant.

## 3. Results

### 3.1. Systemic Effects of Supplementation with Prodefen^®^

**Figure 1:**
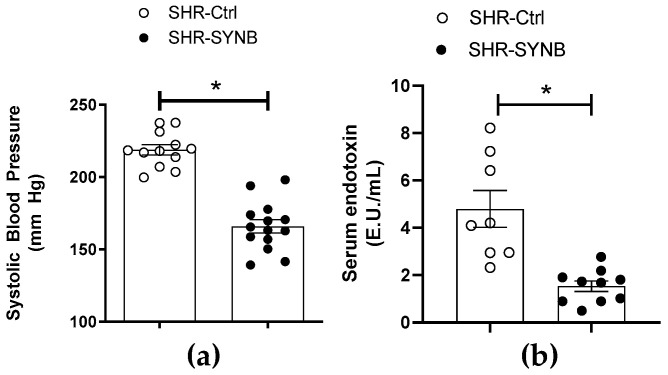
(**a**) Effect of supplementation with Prodefen^®^ on systolic blood pressure. Results (Mean ± S.E.M.) are expressed in mm Hg. *n* = 12–14 animals each group. * *p* < 0.05 (Student’s *t*-test). (**b**) Serum endotoxin levels in SHR-Ctrl and SHR-SYNB. Results (Mean ± S.E.M.) are expressed in Endotoxin units (E.U.)/mL. *n* = 8–10 animals each group. * *p* < 0.05 (Student’s *t*-test).

In the present study we aimed to determine whether supplementation with the synbiotic formulation Prodefen^®^ could reduce high blood pressure in an experimental model of hypertension (spontaneously hypertensive rats), as well as the possible mechanisms implicated in this reduction. All SHR showed hypertension at the beginning of the experimental procedure. Despite four-week supplementation with Prodefen^®^ reduced systolic blood pressure (Figure 1a), the hypertensive phenotype remained in SHR-SYNB animals, since they did not reach the systolic blood pressure values previously described in normotensive Wistar-Kyoto (WKY) rats (Supplementary Materials) [32].

Multiple pathologies, like hypertension, are in part caused by bacterial translocation, which induces an inflammatory and prooxidant phenotype, and gut microbiota modulation can reduce this bacterial translocation. Given the above, we analyzed serum endotoxin levels as a marker of bacterial translocation. We observed that Prodefen^®^ reduced endotoxin serum levels, suggesting that this supplementation avoided bacterial translocation (Figure 1b).

**Figure 2:**
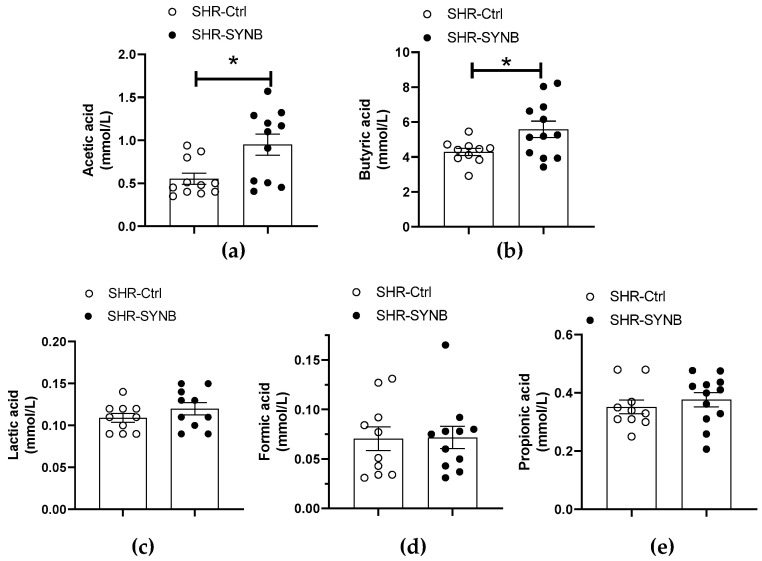
Levels of acetic (**a**), butyric (**b**), lactic (**c**), formic (**d**) and propionic (**e**) acids in fecal samples from SHR-Ctrl and SHR-SYNB animals. Results (Mean ± S.E.M.) are expressed in mmol/L. *n* = 10–12 animals each group. * *p* < 0.05 (Student’s *t*-test).

Among the metabolites released from gut microbiota, SCFA can be absorbed from the colon to the bloodstream, participating in the maintenance of homeostasis. Different physio-pathological situations can modify the production of SCFA, thereby contributing to the development and/or maintenance of the disease. Regarding hypertension, decreases in acetic, propionic and butyric acids have been reported in fecal samples from SHR, when compared with their normotensive control WKY [33]. The modulation of gut microbiota composition by the supplementation with different probiotic, prebiotic or synbiotic agents gave rise to the production of SCFAs. In this sense, when fecal samples from SHR-Ctrl and SHR-SYNB were compared, no significant differences were found in the case of lactic, formic or propionic acids. However, significant increases were detected in acetic and butyric acids between SHR-Ctrl and SHR-SYNB (Figure 2). Therefore, the intake of Prodefen^®^ stimulated the production of SCFA in SHR.

### 3.2. Contractile Response to Electrical Field Stimulation

**Figure 3:**
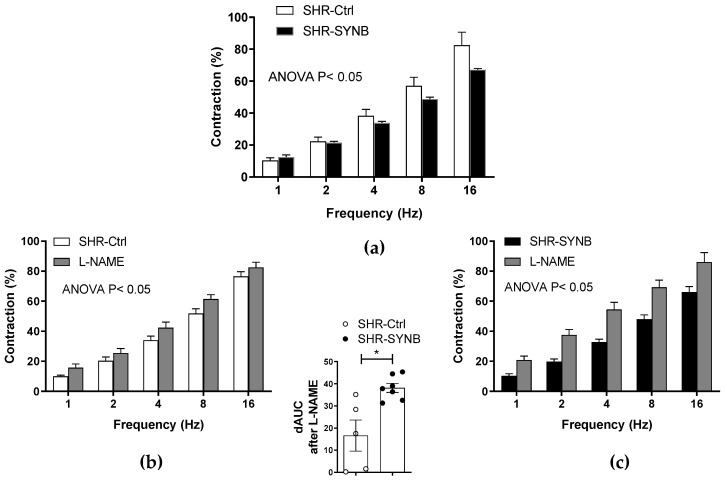
(**a**) Vasoconstriction induced by electrical field stimulation (EFS) in endothelium-denuded mesenteric segments from SHR-Ctrl and SHR-SYNB animals (*n* = 6–8 segments from different rats in each experimental group). Results (mean ± S.E.M.) are expressed as a percentage of the previous tone elicited by KCl. Analysis of the functional role of neuronal NO on EFS-induced vasoconstriction by preincubation with the unspecific nitric oxide synthase (NOS) inhibitor L-NAME, in mesenteric arteries from SHR-Ctrl (**b**) and SHR-SYNB (**c**). Results (mean ± S.E.M.) are expressed as a percentage of previous tone induced by KCl. *n* = 5–6 segments from different animals in each experimental group. Insert panel: Differences in the area under the curve (dAUC) in presence/absence of L-NAME. * *p* < 0.05 SHR-Ctrl vs. SHR-SYNB (Student’s *t*-test).

The alterations in blood pressure are partially linked to modifications in peripheral vascular resistance. Among the multiple vasoactive factors which regulate vascular tone in the superior mesenteric artery, perivascular innervation plays a relevant role [8]. The application of an EFS elicits a contractile response because of the integrated vasomotor response of all the neurotransmitters released from perivascular innervation. The results showed a frequency-dependent contractile response in endothelium- denuded SMA segments from both groups (Figure 3a). This contraction was lower in segments from SHR-SYNB.

### 3.3. Modifications on Nitrergic Component of Mesenteric Innervation

**Figure 4:**
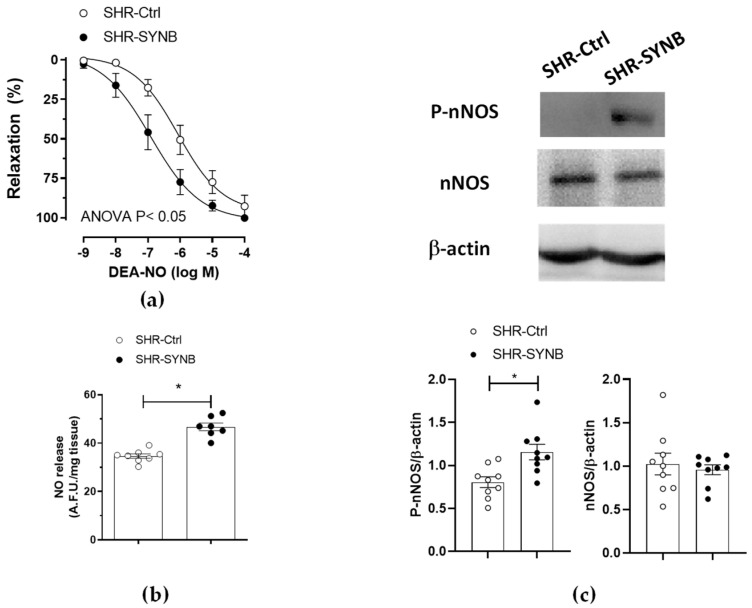
(**a**) Vasodilator response to NO donor DEA-NO in noradrenaline-precontracted mesenteric segments from SHR-Ctrl and SHR-SYNB rats (*n* = 6 segments from different animals in each experimental group). Results are expressed as mean ± S.E.M. (**b**) EFS-induced NO release in mesenteric arteries from SHR-Ctrl and SHR-SYNB. Data (Mean ± S.E.M.) are expressed as arbitrary fluorescence units/mg tissue. *n* = 7–8 segments in each group. * *p* < 0.05 (Student’s *t*-test). (**c**) Analysis of nNOS expression and phosphorylation (Ser 1417) in mesenteric rings from SHR-Ctrl and SHR-SYNB. The figure is representative of 9 isolated segments from each group. Lower panel: Densitometry analysis for the expression of each protein. Results (mean + S.E.M.) are expressed as the relation between the signal obtained for the analyzed protein and the signal obtained for β-actin. * *p* < 0.05 (Student’s *t*-test).

Nitrergic innervation has great relevance in the regulation of mesenteric resistance by releasing the potent vasodilator NO as a neurotransmitter. Although the decrease in NO released from nitrergic innervation participates in the development of hypertension in obesity and metabolic syndrome, classical studies from our group demonstrated an enhanced role of neuronal NO in SHR. In fact, we have previously described that nitrergic innervation did not have a functional role in the normotensive WKY rats (Supplementary Materials), thereby providing a compensatory role for nitrergic innervation in hypertensive rats, that counteracts the increased vascular resistance observed in this pathology [11,12]. Given the lower EFS-induced contraction observed in segments from SHR-SYNB, we aimed to determine the possible alterations in nitrergic innervation function. For this purpose, we preincubated de-endothelized mesenteric rings from both SHR-Ctrl and SHR-SYNB with the non-specific NOS inhibitor L-NAME, (0.1 mmol/L), observing a significant increase in EFS-induced vasoconstriction in arteries from both experimental groups (Figure 3b,c). A further analysis of dAUC (insert panel) showed that this increase was greater in arteries from SHR-SYNB animals. Consequently, we could assume that the supplementation with the commercial synbiotic formulation Prodefen^®^ produced an increase in the participation of nitrergic innervation. In addition, a greater vasodilator response to NO donor DEA-NO was observed in segments from SHR-SYNB animals (Figure 4a).

### 3.4. Mechanisms Implicated in Neuronal Nitric Oxide Release

NO is one of the most important vasoactive factors, due to its vasodilatory effect. In the vascular tissue, NO can be released from endothelial cells, vascular smooth muscle and perivascular innervation. A decrease in endothelial NO release has been widely reported in hypertension, producing endothelial dysfunction and contributing to the increase in vascular resistance observed in this pathology [34]. Regarding neuronal NO, we have previously reported enhanced NO release in mesenteric segments of SHR, compared to their normotensive control WKY (Supplementary Materials) [11]. This result is related to the lack of functional role of neuronal NO in WKY rats and reinforces the hypothesis that neuronal NO release has a compensatory role in mesenteric arteries from SHR. Synbiotic agents have been described to modulate nitrergic innervation function, by enhancing neuronal NO release [12]. We observed that the application of an EFS pattern induced NO release in mesenteric segments without endothelium from both SHR-Ctrl and SHR-SYNB groups. Interestingly, this NO release was greater in segments from SHR-SYNB group. (Figure 4b). This increase can be due either to alterations in nNOS expression and/or activity. We found that the expression of nNOS was comparable among groups, while its phosphorylation was greater in arteries from SHR-SYNB group (Figure 4c).

**Figure 5:**
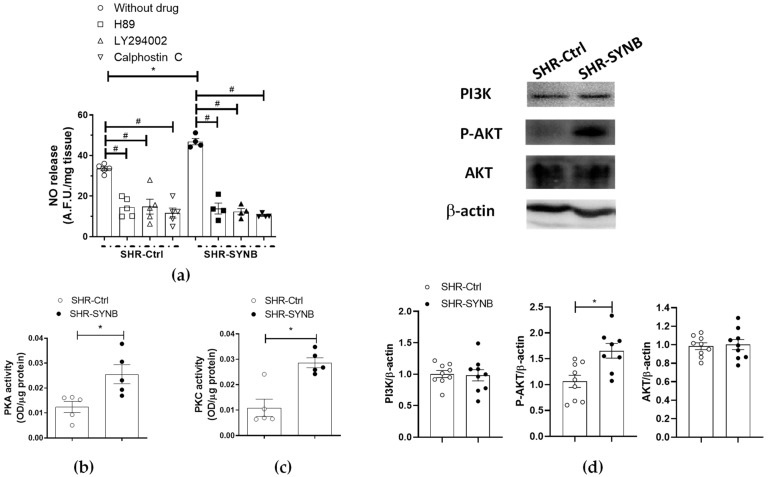
(**a**) Inhibitory effect of H89 (PKA inhibitor, 1 µmol/L), calphostin C (PKC inhibitor, 0.1 µmol/L) or LY 294002 (PI3K inhibitor, 10 µmol/L) on EFS-induced NO release in endothelium-denuded mesenteric rings from SHR-Ctrl and SHR-SYNB (*n* = 4–5 segments in each experimental group). Data (arbitrary fluorescence units/mg tissue) are expressed as mean ± S.E.M. * *p* < 0.05 SHR-Ctrl vs. SHR-SYNB (Student’s *t*-test). # *p* < 0.05 conditions without inhibitor vs. conditions with inhibitor in each group (Student’s *t*-test). (**b**) PKA activity, and (**c**) PKC activity in mesenteric arteries from SHR-Ctrl and SHR-SYNB (*n* = 5 segments from different animals in each group). Results (optical density (OD) units/µg protein) are represented as (mean ± S.E.M). * *p* < 0.05 (Student’s *t*-test). (**d**) Analysis for AKT and PI3K (P85 subunit) expression, and AKT phosphorylation at the T308 residue (P-AKT) in mesenteric arteries from SHR-Ctrl and SHR-SYNB (8–9 isolated arterial segments from different animals in each group). Lower panel: Densitometry analyses of the protein expression. Results (mean ± S.E.M) are expressed as protein expression relative to β-actin expression. * *p* < 0.05 (Student’s *t*-test).

PKA, PKC and PI3K/AKT signaling pathways play a crucial role in the activation of nNOS. To analyze the involvement of these pathways in our experimental conditions, we used specific pharmacological inhibitors before EFS-stimulation and measurement of NO release. Thus, we observed that the PKA inhibitor H89 (1 µmol/L) decreased EFS-induced NO release to a greater extent in arteries from rats supplemented with Prodefen^®^ (Subtraction, in A. F. U.: SHR-Ctrl: 19.01 + 2.4; SHR-SYNB: 29.74 + 3.9; *p* < 0.05; % of inhibition: SHR-Ctrl: 54.9+ 6.9; SHR-SYNB: 70.6 + 2.9; *p* = 0.099). Similar results were found after preincubation with LY394002 (10 µmol/L), a PI3K inhibitor (Subtraction, in A. F. U.: SHR-Ctrl: 18.71 + 2.9; SHR-SYNB: 34.52 + 0.7; *p* < 0.05; % of inhibition: SHR-Ctrl: 63.2 + 4.9; SHR-SYNB: 77.9 + 3.4; *p* = 0.074) or with the PKC inhibitor Calphostin C (0.1 µmol/L) (Subtraction, in A. F. U.: SHR-Ctrl: 21.9 + 1.7 %; SHR-SYNB: 36.4 + 1.6; *p* < 0.05; % of inhibition: SHR-Ctrl: 53.9 + 8.3; SHR-SYNB: 73.9 + 1.5; *p* = 0.0504). Altogether, these results suggest that the three signaling pathways could be responsible for the greater nNOS phosphorylation and, consequently, the major NO release in arteries from SHR-SYNB animals. This hypothesis was confirmed by the fact that PKA and PKC activities, and AKT phosphorylation were greater in arteries from rats treated with Prodefen^®^ (Figure 5).

### 3.5. Neuronal Nitric Oxide Bioavailability: Oxidative Stress

**Figure 6:**
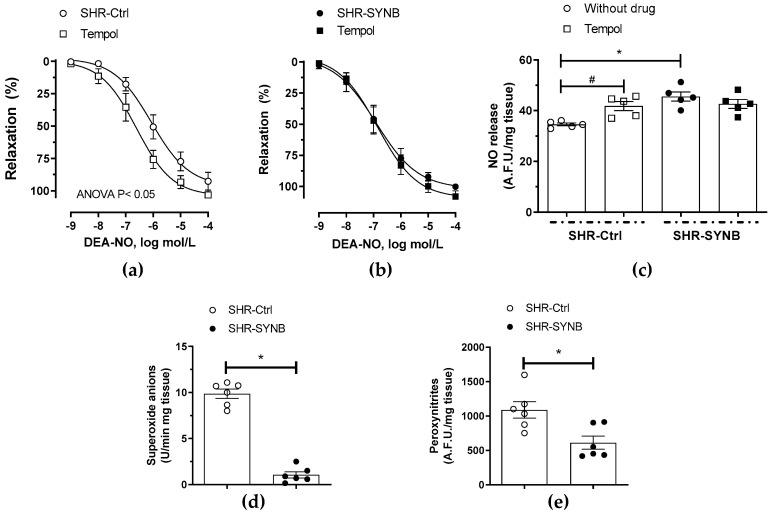
Effect of preincubation with 0.1 mmol/L tempol (a superoxide anion tempol) on the vasodilator response to NO donor DEA-NO in mesenteric segments from SHR-Ctrl (**a**) and SHR-SYNB (**b**). Results (mean ± S.E.M.) are expressed as a percentage of the previous tone elicited by noradrenaline. *n* = 6 segments from different animals in each experimental group. (**c**) Effect of preincubation with tempol on EFS-induced NO release in mesenteric arteries from SHR-Ctrl and SHR-SYNB. Data (Mean ± S.E.M.) are expressed as arbitrary fluorescence units/mg tissue. * *p* < 0.05 SHR-Ctrl vs. SHR-SYNB (Student’s *t*-test). # *p* < 0.05 conditions without tempol vs. conditions with tempol in each group (Student’s *t*-test). *n* = 5 segments in each experimental group. (**d**) Superoxide anion formation in mesenteric segments from SHR-Ctrl and SHR-SYNB. Results (mean ± S.E.M.) are expressed as chemiluminiscence units (U)/min mg tissue. *n* = 6 segments in each group. *p* < 0.05 (Student’s *t*-test). (**e**) EFS-induced peroxynitrite release in mesenteric arteries from SHR-Ctrl and SHR-SYNB. Data (Mean ± S.E.M.) are expressed as arbitrary fluorescence units/mg tissue. *n* = 6 segments in each group * *p* < 0.05 (Student’s *t*-test).

Oxidative stress can modulate NO function by diminishing its bioavailability. In line with this, we observed that preincubation with 0.1 mmol/L tempol (a superoxide anion scavenger) enhanced both NO release and vasodilator response to NO donor DEA-NO in arteries from SHR-Ctrl group, while it had no effect in segments from SHR-SYNB animals (Figure 6a–c). These results suggest that treatment with Prodefen^®^ exerted an antioxidant role, which was confirmed by the fact that both superoxide anion and EFS-induced peroxynitrite releases were lower in arteries from SHR-SYNB animals (Figure 6d,e).

**Figure 7:**
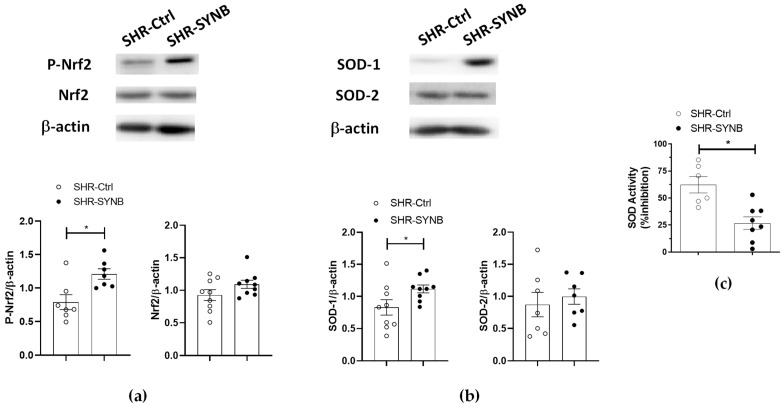
Western blot analysis for (**a**) total Nrf2, phosphorylated Nrf2 in Ser40 residue (P-Nrf2), and (**b**) SOD-1 and SOD-2 in mesenteric segments from SHR-Ctrl and SHR-SYNB. (7–9 isolated arterial segments from different animals in each group). Lower graphs show densitometry analyses of the protein expression. Results (mean ± S.E.M) are expressed as protein expression relative to β-actin expression. * *p* < 0.05 (Student’s *t*-test). (**c**) Superoxide dismutase activity in mesenteric segments from SHR-Ctrl and SHR-SYNB. Results (mean ± S.E.M.) are expressed as a percentage of inhibition (% inhibition). *n* = 6-8 segments in each group. * *p* < 0.05 (Student’s *t*-test).

Among the antioxidant defense mechanisms present in the organism, transcription factor Nrf2 regulates the adaptive response to oxidative stress by translocating to the nucleus, due to its phosphorylation, and promoting transcription of antioxidant enzymes, like SOD. In our experimental conditions we observed no differences in total Nrf2 expression, while its phosphorylation was greater in arteries from animals supplemented with Prodefen^®^ (Figure 7a). Accordingly, a higher expression of SOD-1 in SHR-SYNB was observed, while SOD-2 expression was not modified (Figure 7b). In addition, the percentage of inhibition of SOD was greater in arteries from SHR-SYNB group, confirming greater antioxidant activity after preincubation with Prodefen^®^ (Figure 7c).

## 4. Discussion

Hypertension is one of the most prevalent diseases worldwide, being a precursor of numerous cardiovascular diseases, which are the leading cause of death globally [3]. This multifactorial pathology is characterized by maintained enhancement in systemic blood pressure. Alterations in GM composition have been proven to be implicated in the pathogenesis of hypertension [21], and modulation of gut microbiota, by supplying synbiotic agents, can promote recovery of blood pressure levels to normotensive values [1,2,3,4,5]. In line with this, we previously demonstrated, in a metabolic syndrome model, that supplementation with a commercial synbiotic formulation reversed hypertension to normotensive levels [12].

Regarding the above, our first objective was to determine whether the modulation of gut microbiota with the commercially available synbiotic formulation Prodefen^®^ for 4 weeks could ameliorate hypertension in SHR. We observed a decrease in systolic blood pressure after supplementation with Prodefen^®^ for 4 weeks. It is noteworthy that in the present study we are using a genetical model of hypertension. For that reason, the reduced systolic blood pressure observed after supplementation with Prodefen^®^ did not reach the normotensive values previously described in Wistar-Kyoto (WKY) rats, as it happened in a previously used model of diet-induced hypertension [12,32]. These results can also be extrapolated to humans. To the best of our knowledge, no clinical trial with Prodefen^®^ has been conducted to determine possible improvement in hypertension. Despite this, different meta-analyses have been recently published, showing reductions in systolic blood pressure in hypertensive patients supplemented with different synbiotic formulations containing fructooligosaccharides, and different *Lactobacillus*, *Streptococcus* and *Bifidobacterium* strains, similar to Prodefen^®^ [35,36,37]. Consequently, supplementation with synbiotic agents can ameliorate hypertension in both animal models and humans.

An important link exists between hypertension and inflammation. In fact, different hypertensive animal models, as well as patients, present elevated levels of proinflammatory cytokines, adhesion molecules and inflammatory enzymes in different tissues including the vasculature [38,39,40]. Both gut microbiome and its metabolites have been implicated in the regulation of host physiological functions, such as inflammatory and metabolic responses. Thus, the translocation of isolated microbial products, such as endotoxin or bacterial DNA, has also been associated with the development of systemic inflammation. In fact, bacterial LPS translocation to the bloodstream can induce a low-grade vascular inflammatory phenotype, thereby contributing to the rise of blood pressure in hypertension [41]. Previous studies have reported that supplementation with different probiotic strains can prevent this endotoxemia present in SHR [42]. These findings were confirmed in our experimental conditions after supplementation with the synbiotic formula Prodefen^®^.

Among the metabolites released from gut microbiota, SCFA are produced from indigestible carbohydrates, such as dietary fibers, and can be absorbed from the colon to the bloodstream. Relevant SCFA increases were observed during fermentation of prebiotics. Increases observed for butyric and acetic acid are in accordance with the literature, as major end-products of saccharolytic fermentation are acetate, butyrate and propionate [43]. These SCFA are absorbed into the bloodstream through colonic vasculature and can have different systemic effects, which can interfere with blood pressure. SCFAs can join to different G-coupled receptors in both vascular and renal tissue, with opposite effects. GPR41 receptor can be found in both smooth muscle and endothelial cells, and its absence in knock-out mice has been linked to greater blood pressure and a higher cardiac hypertrophy index [44]. In addition, signaling through this receptor in Treg lymphocytes can activate different anti-inflammatory pathways, protective in different hypertension models [45,46,47]. On the other hand, the olfactory receptor Olfr78, found in renal tissue, can join both acetate and propionate, stimulating the secretion of renin and consequent activation of the renin angiotensin aldosterone system. The lack of this receptor has a hypotensive effect [48,49]. In summary, high levels of SCFA are desirable due to their relationship with beneficial effects on human health, exerting both direct and indirect effects on vasculature and, consequently, modulating vasodilation and reducing blood pressure. Different studies performed in hypertensive humans and animal models of hypertension have reported that supplementation with different probiotic or synbiotic agents enhances acetate, butyrate, and propionate, leading to an amelioration of hypertension [50,51]. In addition, different authors observed significant increases in acetic, butyric and propionic acids after ingestion of fructooligosaccharides in different animal models, including SHR animals [52,53,54,55]. Regarding our study, we observed increases in acetic and butyric acid after supplementation with Prodefen^®^. Consequently, and, as suggested by Robles-Vera et al. [51], this increase in SCFA might be related to reduction of vascular resistance, thereby participating in reduction in blood pressure levels.

One of the pivotal causes of the development of hypertension is an increase in peripheral vascular resistance. The mesenteric vascular bed, located in the peritoneal cavity, belongs to the splanchnic vasculature, and plays a major role in generating and maintaining systemic vascular resistance. The vascular tone in this artery is regulated by multiple endothelial, myogenic, hormonal, and neuronal factors. We have previously demonstrated that alterations in the perivascular innervation function are of great relevance in the onset and maintenance of cardiovascular disturbances in multiple pathophysiological situations, including hypertension [11,12,13,15,19]. In addition, acute incubation with LPS, which mimics a proinflammatory microenvironment, enhanced vasoconstriction induced by perivascular innervation [30]. Previous studies have demonstrated improvement of endothelium-dependent vasodilation in hypertension after gut microbiome modulation with probiotic agents [56,57]. In addition, we also proved an improvement in mesenteric neuronal function in metabolic syndrome animals treated with a synbiotic formula [12]. Altogether, these evidences led us to determine whether the reduction in blood pressure observed in SHR-SYNB animals could be partly due to amelioration of the perivascular innervation function. For that purpose, we applied an EFS pattern to endothelium-denuded segments from both SHR-Ctrl and SHR-SYNB animals and observed a lower frequency-dependent vasoconstriction in Prodefen^®^-supplemented rats. Given the fact that the vasoconstrictor response to KCl was similar in arteries from both experimental groups, we can rule out possible alterations in the contractile machinery due to supplementation with Prodefen^®^. Consequently, these alterations can be due to modifications in the participation of perivascular mesenteric innervation.

Among the components of mesenteric innervation, sympathetic and nitrergic innervations play a relevant role. Sympathetic hyperactivity plays a relevant role in the origin and maintenance of hypertension, due to enhanced release of, or vasomotor response to, the neurotransmitter noradrenaline and/or the co-transmitter ATP [7,8]. A recent review from Robles-Vera et al. [58] summarizes the role of dysbiosis in hypertension. According to several authors, sympathetic overactivation leads to the development of dysbiosis [59,60,61], and the modulation of gut microbiota through fecal transplantation reduces this sympathetic dysfunction, thereby showing possible crosstalk between hyperactivation of the sympathetic nervous system and dysbiosis in hypertension [60,62].

Regarding nitrergic innervation, the vasodilator neurotransmitter NO can reduce the maximum tone generated by noradrenaline by up to 50% [7,8]. Neuronal NO release is altered in situations in which vascular resistance is modified. In fact, although the decrease in NO released from nitrergic innervation participates in the development of hypertension in obesity and metabolic syndrome, classical studies from our group demonstrated an enhanced role of neuronal NO in SHR. Given the fact that nitrergic innervation did not have a functional role in the normotensive WKY rats, we assumed that the enhanced role for the nitrergic component observed in SHR had a compensatory role for nitrergic innervation, thereby counteracting the increased vascular resistance observed in this pathology [10,11,12,19]. A great number of studies have determined that supplementation with different probiotic or synbiotic formulations re-established both endothelial and neuronal function in different pathologies which might participate in restoring blood pressure [12,57]. Accordingly, here we found that supplementation with Prodefen^®^ could increase the participation of neuronal NO in EFS-induced vasoconstriction.

We have previously demonstrated that alterations on nitrergic participation in SMA in different pathophysiological situations can be due to either NO release and/or NO-dependent vasodilation [13,15,19]. Regarding the former, we observed an enhanced vasodilator response to exogenous NO, and an increase in EFS-induced NO release in segments from rats submitted to metabolic syndrome, supplemented with a commercial synbiotic formulation. Similar results regarding endothelial NO release were reported after modulation of gut microbiota with either probiotic or synbiotic agents [12,24,56]. In line with this, we found greater NO release from nitrergic nerve terminals in segments from SHR-SYNB animals. This result reinforces the fact that gut microbiota disturbances can modify nitrergic regulation of vascular tone, in this case by altering the release of neuronal NO.

nNOS is the enzyme responsible for the synthesis of NO in nitrergic innervation. We have reported increases, decreases or no modifications in mesenteric nNOS expression in different pathologies that manifest hypertension [13,19,31]. Several studies have demonstrated that modulation of gut microbiota can increase expression of different constitutive NOS isoforms in different tissues [63,64,65], which can lead to an enhanced NO release. However, in this study we found no differences in nNOS expression between arteries from SHR-Ctrl and SHR-SYNB groups. Previous studies have showed either directly or indirectly an increase in the activity of both constitutive eNOS and nNOS isoforms after supplementation with different probiotic or synbiotic formulations [12,24]. The fact that we found a greater degree of phosphorylation on Ser1417 residue of nNOS in arteries from SHR-SYNB animals confirmed greater nNOS activity after supplementation with Prodefen^®^, that would explain the increase in NO release observed in this experimental group.

Different kinases, such as PKA, PKC or PI3K/AKT pathways are essential for multiple physiological responses. Alterations in these pathways have been reported in different hypertensive models [11,66,67,68]. In this study, we observed that supplementation with Prodefen^®^ enhanced PKA activity, similar to that described in a metabolic syndrome experimental model [12]. In addition, both PKC activity and AKT phosphorylation and subsequent activation were also increased in mesenteric segments from SHR-SYNB animals. Even though only a few inconsistent results were reported regarding the effect of probiotic and synbiotic agents on the signaling pathways activated by those kinases [69,70,71,72,73,74,75], their role in phosphorylation and subsequent activation of nNOS is well demonstrated [10,13,76]. We observed that the pharmacological inhibition of PKA, PKC and PI3K with H89, calphostin C or LY294002, respectively, diminished EFS-induced NO release in arteries from both SHR-Ctrl and SHR-SYNB groups. These decreases were greater in animals supplemented with Prodefen^®^, correlating with the enhanced activity of these kinases. Overall, we can conclude that supplementation with the commercial formulation Prodefen ^®^ might be able to ameliorate hypertension by enhancing PKA, PKC and PI3K-AKT activities, thereby producing neuronal NO over-release in rat mesenteric arteries, and potentiating, in turn, the participation of perivascular nitrergic innervation.

The results described above could explain by themselves the increase on nitrergic function due to supplementation with Prodefen^®^. However, aside from its release, it is also important to determine the bioavailability of NO, which depends on oxidative stress. Reactive oxygen species (ROS) have been shown to play a critical role in hypertrophy, fibrosis, and remodeling in the heart and vasculature [77,78,79]. An enhanced superoxide anion formation has also been demonstrated in hypertension, because of the action of different mechanisms [14,80]. The pro-oxidative microenviroment that appears in hypertension might be implicated in the alterations in nitrergic function, producing an increase in oxidative stress and, consequently, blunted NO bioavailability. In line with this, our group have reported an increase in superoxide anion release in obesity and hypertension [15,81], which reacts with NO, producing peroxynitrite [30,31]. Several groups have revealed beneficial effects of different probiotic and synbiotic agents due to their reduction of oxidative stress in different tissues, including vascular tissue [56,82,83,84,85]. These reports agree with the reduction in both superoxide anion and peroxynitrite that we observed in arteries from SHR-SYNB animals. The reduced mesenteric oxidative stress that we found has a functional repercussion; in that the preincubation with the superoxide anion scavenger tempol increased vasodilator response in segments from SHR-Ctrl animals, while it had no effect in mesenteric rings from the SHR-SYNB group. Similarly, the EFS-induced NO release was enhanced by tempol only in SHR-Ctrl animals.

The organism contains multiple antioxidant defense mechanisms, which keep oxidative stress to the appropriate levels. Regarding superoxide anion, the main enzyme responsible for its physiological metabolism is SOD, the activity and expression of which are reduced in the cardiovascular system in hypertension [86,87]. Regarding several studies, manipulation of gut microbiota with different approaches, including supplementation with synbiotics and probiotics, restored these alterations [88,89,90]. In our experimental conditions we observed greater SOD activity in arteries from rats supplemented with Prodefen^®^, thereby explaining the diminished vascular oxidative stress that we observed in SHR-SYNB rats. There are several SOD isoforms that could be implicated in this increased activity, SOD -1and SOD-2 being present in this vascular bed [76]. Further analysis showed that this increased SOD activity was produced by enhanced SOD-1 expression in arteries from SHR-SYNB animals. In addition, we observed a similar SOD-2 expression in both experimental groups, suggesting that this isoform might not have a repercussion in the increased SOD activity observed in SHR-SYNB animals.

Transcription factor Nrf2 regulates the adaptive response to oxidative stress. In situations in which ROS levels are increased, Nrf2 is activated, either by dissociation from the regulator subunit Keap1, or by phosphorylation by the action of various kinases, such as PI3K/AKT or PKC. Therefore, Nrf2 translocates to the nucleus, promoting the transcription of different genes with an antioxidant role i.e., SOD [16,17,18]. Diminished levels of Nrf2 and the antioxidant enzymes regulated by this transcription factor have been observed in hypertension [18]. Several reports have shown the beneficial role of probiotic supplementation in increasing the activity of various antioxidant enzymes at the systemic level [23,24], restoring NO to physiological levels. The increased SOD-1 expression that we observed led us to study whether supplementation with Prodefen^®^ could increase in the Nrf2-antioxidant enzyme pathway. First, we observed that the expression of Nrf2 was similar in both experimental groups, contrasting with previous reports [64,91,92], while we found a greater phosphorylation of this transcription factor in arteries from SHR-SYNB animals. The different experimental models (heart, gastric mucosa, or cell cultures) or supplementation (probiotics and prebiotics) used could explain this discrepancy. In addition, we cannot forget that, among the activation mechanisms of Nrf2, phosphorylation through PKA, PKC and PI3K-AKT signaling pathways has been described, and we have observed that Prodefen^®^ enhanced vascular activities of these three pathways. Altogether, supplementation with Prodefen^®^ increased neuronal NO bioavailability, through increased Nrf2/SOD-1 activation, and reduced vascular oxidative stress.

## 5. Conclusions

Overall, our data describe the beneficial effect of Prodefen^®^ in ameliorating high blood pressure in a genetic model of spontaneously hypertensive rats, by enhancing perivascular nitrergic function in the superior mesenteric artery. Two mechanisms are implicated in this blood pressure improvement: 1) increased neuronal NO release through nNOS activation, and 2) enhanced antioxidant effect, mediated by increased Nrf2/SOD-1 activation. Enhanced PKC, PKA and PI3K/AKT activities might be responsible for these alterations. In conclusion, the commercially available formula Prodefen^®^ could be considered an interesting non-pharmacological approach to reduce hypertension.

## Data Availability

Data is contained in the article and supplementary materials.

## Supplementary Materials

The following supporting information can be downloaded at: https://www.mdpi.com/article/10.3390/antiox11040680/s1, Figure S1: (a) Systolic blood pressure from WKY, SHR-Ctrl and SHR-SYNB animals (Mean + S.E.M.; n = 11–14 animals each group) expressed in mm Hg. One-way ANOVA, followed by a Tukey post-hoc test was used as a statistical analysis. * *p* < 0.05 WKY vs. SHR-Ctrl; # *p* < 0.05 SHR-Ctrl vs. SHR-SYNB. + *p* < 0.05 WKY vs. SHR-SYNB. (b) EFS-induced NO release in mesenteric segments from WKY, SHR-Ctrl and SHR-SYNB animals (Mean + S.E.M.; *n* = 11–14 animals each group) expressed in arbitrary fluorescence units (A.F.U.)/mg tissue. One-way ANOVA, followed by a Tukey post-hoc test was used as a statistical analysis. * *p* < 0.05 WKY vs. SHR-Ctrl; # *p* < 0.05 SHR-Ctrl vs. SHR-SYNB. + *p* < 0.05 WKY vs. SHR-SYNB (c) Analysis of the functional role of neuronal NO on EFS-induced vasoconstriction by preincubation with the unspecific nitric oxide synthase (NOS) inhibitor L-NAME, in mesenteric arteries WKY rats. Results (mean ± S.E.M.) are expressed as a percentage of previous tone induced by KCl. *n* = 6 segments from different animals in each ex-perimental group.
