# Supplementary material for: Supplementation with the Symbiotic Formulation Prodefen® Increases Neuronal Nitric Oxide Synthase and Decreases Oxidative Stress in Superior Mesenteric Artery from Spontaneously Hypertensive Rats
Source: Antioxidants (Basel). 2022 Mar 30;11(4):680. doi: 10.3390/antiox11040680 (PMC9029967; doi:10.3390/antiox11040680)
Supplement: Supplementary file 1 [file antioxidants-11-00680-s001.zip › antioxidants-1621025-supplementary.pdf]

Supplementary Materials

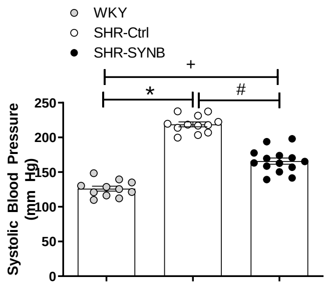

(a)

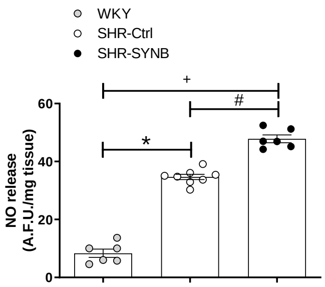

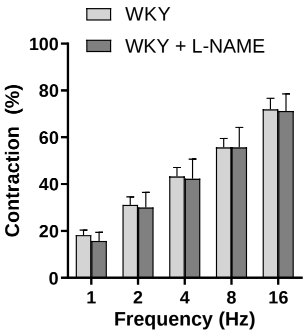

(b) **(c)**

Figure S1. (**a**) Systolic blood pressure from WKY, SHR-Ctrl and SHR-SYNB animals (Mean + S.E.M.; n= 11-14 animals each group) expressed in mm Hg. One-way ANOVA, followed by a Tukey post-hoc test was used as a statistical analysis. * $p< 0.05$ WKY vs. SHR-Ctrl; # $p< 0.05$ SHR-Ctrl vs. SHR-SYNB. + $p< 0.05$ WKY vs. SHR-SYNB. (**b**) EFS-induced NO release in mesenteric segments from WKY, SHR-Ctrl and SHR-SYNB animals (Mean + S.E.M.; n = 11–14 animals each group) expressed in arbitrary fluorescence units (A.F.U.)/mg tissue. One-way ANOVA, followed by a Tukey post-hoc test was used as a statistical analysis. * $p< 0.05$ WKY vs. SHR-Ctrl; # $p< 0.05$ SHR-Ctrl vs. SHR-SYNB. + $p< 0.05$ WKY vs. SHR-SYNB (**c**) Analysis of the functional role of neuronal NO on EFS-induced vasoconstriction by preincubation with the unspecific nitric oxide synthase (NOS) inhibitor L-NAME, in mesenteric arteries WKY rats. Results (mean ± S.E.M.) are expressed as a percentage of previous tone induced by KCl. n = 6 segments from different animals in each experimental group.
